# Quantitative summarization of high-touch surfaces and epidemiological parameters of *Clostridioides difficile* acquisition and transmission for mathematical modeling: a systematic review

**DOI:** 10.1017/ice.2025.10302

**Published:** 2025-12

**Authors:** Isaac Olufadewa, Harrison Latimer, Haleigh N. West-Page, Shi Chen

**Affiliations:** 1 Department of Epidemiology and Community Health, University of North Carolina at Charlotte, Charlotte, NC, USA; 2 Department of Mathematics and Statistics, University of North Carolina at Charlotte, Charlotte, NC, USA

## Abstract

**Objective:**

The study aimed to summarize estimates of key epidemiological parameters to improve the effectiveness of *Clostridioides difficile* infection (CDI) mathematical models and quantitatively characterize high-touch surfaces (HTSs) and mutual-touch surfaces in healthcare settings.

**Methods:**

We systematically searched four databases and applied predefined eligibility criteria to screen, select, and include peer-reviewed studies in accordance with the Preferred Reporting Items for Systematic Reviews and Meta-Analyses guidelines. The study is registered in the International Prospective Register of Systematic Reviews (CRD42023408483).

**Results:**

Among the 21 *C. difficile* infection modeling studies, 76.2% used compartmental model approaches that group patients into infection disease categories such as susceptible, infected, or recovered, while 23.8% applied agent-based model approaches that simulate individual patients, staff, or surfaces. Key epidemiological parameters varied widely: estimates of how many new cases one patient could cause—the basic reproduction number (R₀)—ranged from 0.28, suggesting limited hospital spread, to as high as 2.6, which implies sustained in-hospital transmission. Incubation periods were reported between 4 and 18 days. Recovery and recurrence rates also differed across studies. Quantitative HTSs ranking revealed that bed rails, bedside tables, and supply carts were the top three most frequently touched surfaces.

**Conclusions:**

Our findings highlight that modeling studies used different assumptions and estimates, creating variations in results. Clinicians should interpret modeling outputs, such as predicted spread or effectiveness of an intervention carefully, as differences may reflect real-world variation between hospitals or methodological variation. Developing infection models that reflect real-world conditions will enable healthcare teams better simulate and prioritize interventions, optimize cleaning protocols, and improve CDI transmission models for more targeted prevention.

## Introduction

Nearly 700,000 people in the United States acquire healthcare-associated infections (HAIs) annually, making HAIs a significant threat to patient, clinician, and public health.^1–5^ *Clostridioides difficile* infections (CDI) cause about 30,000 U.S. deaths annually, with outbreaks reported in hospitals, nursing homes, and ICUs; spread mainly via the fecal-oral route, its spores persist and require bleach and soap-and-water handwashing for control.^6–9^ In recent decades, the emergence of new and more virulent *C. difficile* strains (such as the epidemic B1/NAP1/027 strain) has driven outbreaks and heightened public health efforts, yet key uncertainties in transmission dynamics persist, challenging effective prevention and control.^10–14^

Mathematical models (infection models) simulate how pathogens spread in healthcare settings—such as understanding how sporadic cases become epidemics—and inform the control of HAIs such as *C. difficile*. These models which are of different types (Table 1) provide useful insights to support hospital-based and public health decision-making by suggesting potential trends in disease outbreaks and exploring the projected impact of various interventions.^15,16^ While not a substitute for clinical evidence, infection models can complement existing approaches by informing resource allocation, identifying plausible intervention strategies, and simulating “what-if” scenarios in complex healthcare settings. These models can incorporate economic analyses to assess cost-effectiveness, but as simplified versions of real-world settings, they should be interpreted cautiously. However, the accuracy and utility of these infection models, depend heavily on the reliability of the epidemiological parameters used.^17^

**Table 1.** Definitions and practical applications of model types used to simulate *C. difficile* transmission in healthcare settings

Model type	Definition	Key characteristics	Practical application in healthcare epidemiology
Compartmental model	Divides the patient or population group into discrete clinical states (e.g., susceptible, colonized, infected) and models transitions between them using differential equations.	Assumes homogeneous mixing; estimates average behavior across groups; widely used for forecasting.	Estimates average rates of *C. difficile* acquisition among patients under different infection control interventions.
Agent-based model (ABM)	Simulates the behavior of individual ‘agents’ (e.g., patients, healthcare workers, or surfaces) and tracks their interactions over time.	Captures individual variability; useful for modeling transmission within complex environments (e.g., wards).	Evaluates how patient–staff–fomite interactions contribute to pathogen spread in an ICU or medical unit.
Stochastic model	Incorporates random variation into transmission processes to reflect real-world uncertainty and variability in infection dynamics.	Accounts for chance events and variability; more realistic in small populations or outbreak settings.	Assesses the probability of transmission during patient or surface contact given uncertain environmental contamination.
Deterministic model	Uses fixed parameter values to generate the same outcome for each simulation run, without accounting for random variation.	Predictable; best suited for exploring average outcomes or comparing policy scenarios at scale.	Compares outcomes of universal decolonization vs. targeted isolation strategies under idealized assumptions.
Hybrid model	Combines two or more modeling approaches (e.g., agent-based and stochastic model; compartmental and deterministic model) to balance realism and scalability.	Offers flexibility to simulate individual-level complexity while retaining population-level insights.	Simulates ward-level prevalence trends while capturing detailed staff-patient interactions and fomite contacts.

Evidence has also shown that fomites, such as environmental surfaces and medical devices, play a critical role in the transmission of HAIs.^18–23^ Several studies, including a systematic review and meta-analysis, have found that patients admitted to wards where a previous patient had *C. difficile* face an increased risk of acquiring CDI compared to those admitted to a ward with no such history.^24–30^ However, there have been limited studies quantifying high-touch surfaces (HTSs).^31,32^ Many earlier studies on the role of environmental surfaces and HTS were qualitative, relying on anecdotal experience, expert knowledge, and assumptions—such as the expectation that objects and surfaces frequently contacted by patients would be the most touched surfaces.^33,34^

This study identifies and summarizes quantitative estimates of key epidemiological parameters to improve the effectiveness of *C. difficile* infection models. Second, we quantitatively characterize HTS and mutual-touch surfaces in healthcare settings by analyzing contact frequency, contact duration, and temporal variations in contact episodes.

## Methods

### Literature search strategy and study selection process

We searched four major literature databases—Web of Science, PubMed, Cumulative Index to Nursing and Allied Health Literature (CINAHL), and the Cochrane Database for Systematic Reviews—from the inception of each database until July 8, 2023, for our first objective (*C. difficile* mathematical modeling parameters) and June 30, 2023, for our second objective (quantifying HTSs). A reference search was also conducted. Additionally, we reviewed gray literature and relevant sources from public health organizations, including the World Health Organization (WHO) website, for both systematic reviews.

For the first systematic review, the search was conducted using Medical Subject Headings (MeSH) and Boolean operators, incorporating three groups of keywords:

1. Mathematical modeling (e.g., “Model*” OR “Mathematical”)
2. *C. difficile* (e.g., “*Clostridioides*” OR “*Clostridium difficile*”)
3. Epidemiological parameters (e.g., “Transmission Coefficient” OR “Recovery Rate”)

For the second systematic review, the search queries included terminologies and synonyms related to major HAI concepts. The keywords and key phrases were categorized into four groups:

1. Fomites, including a list of potential fomites identified from prior literature (e.g., computer, bed rail, supply cart).
2. Contact patterns, including contact frequency, contact duration, and shared-touch patterns (e.g., “high-touch,” “low-touch,” “mutual-touch”).
3. Specific major HAI pathogens, including *C. difficile*, methicillin-resistant *Staphylococcus aureus*, among others.
4. Types of healthcare facilities, such as “medical ward” and “surgical ward.”

The complete search strategy for both systematic reviews is provided in Supplementary Document 1.

### Inclusion and exclusion criteria for review

For the first review objective, we included mathematical modeling studies that provided evidence on the acquisition and/or transmission of *C. difficile* and reported at least one infection modeling parameter. We excluded literature reviews, letters to the editor, commentaries, books, and online reports.

For the second review objective, we included studies that met the following criteria: (1) utilized an observational study design, (2) provided quantitative information on at least one of the three study objectives, and (3) were either peer-reviewed journal articles or documents from reputable organizations such as the CDC or WHO. We excluded studies that relied on anecdotal evidence, common knowledge, or expert opinions regarding contact patterns in healthcare settings. Additionally, we excluded qualitative studies that lacked numerical data, non-peer-reviewed documents (except those from reliable sources), books, and summative literature reviews.

### Screening process

**Fig. 1.**
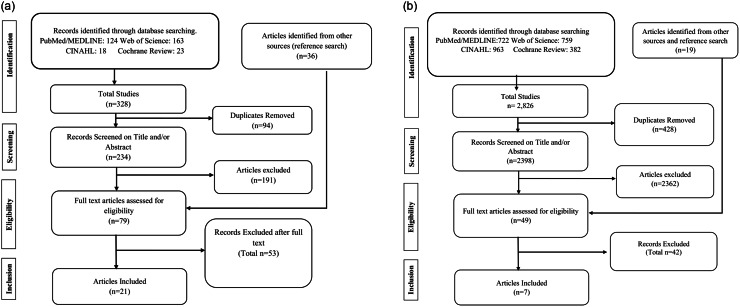
**A** PRISMA flow diagram from the screening process to identify eligible studies for systematic review on the epidemiological parameters of *C. difficile* (Objective 1). **b** PRISMA flow diagram of the systematic review on the quantitative summarization of high-touch surface studies (Objective 2).

For both systematic review objectives, we conducted a title and abstract screening to identify studies that met the inclusion criteria. Abstract screening was performed after title screening to allow for a more in-depth evaluation. Studies deemed relevant after abstract screening were retrieved for a full-text review and data extraction. Further details of the screening process are provided in the Preferred Reporting Items for Systematic Reviews and Meta-Analyses (PRISMA) flow diagrams (Fig. 1a and 1b).

### Data extraction and management

We extracted relevant information from the final set of included studies following the screening process. For the first systematic review, the extracted data were categorized into two major groups. The first category was metadata, which included publication details such as title, authors, and year of publication. The second category was model-related data, including the structure of the infection model which refers to how patients, staff and surfaces are represented: compartmental models group individuals into infection disease categories (e.g., susceptible, infected, recovered) during their simulation of pathogen spread, while agent-based models track individual “agents” (such as patients or staff) and their interactions during their simulation process. Another aspect is the dynamics of the model refer to how uncertainty and variability are handled: deterministic models produce the same result for the same inputs, stochastic models incorporate randomness to reflect real-world variation, and hybrid models combine both approaches. This distinction helps clinicians understand both the level of detail and the predictability of model predictions for infection control planning.

For the second systematic review, the extracted information included study details such as author list, study period, study design, study duration, major findings, HAI pathogens, list of fomites and/or surfaces, contact frequency, and contact duration, among other relevant factors. For the overall ranking of HTS, we calculated a cumulative total ranking score by averaging rankings from three or more healthcare settings in which HTS were quantitatively assessed. This was achieved by dividing the cumulative total ranking score of each surface by the number of studies that provided quantitative ranking data. The entire screening process for both systematic reviews was managed using EndNote software, version 20.

### Systematic review registration

This study was registered in the International Prospective Register of Systematic Reviews (PROSPERO) under registration number CRD42023408483, in accordance with international standards for conducting and reporting systematic reviews. We followed the guidelines of the Cochrane Collaboration and the PRISMA.

## Results

For the first systematic review, we identified 328 initial studies from four major databases that matched our search criteria. The distribution of studies across the four databases was as follows: Web of Science (163), PubMed (124), Cochrane Review (23), and CINAHL (18). After deduplication and full-text screening, 21 studies met the eligibility criteria.

The 21 included *C. difficile* mathematical modeling studies that met our study protocol eligibility criteria included, based on 16 studies (76.2%) used compartmental modeling approaches that group patients into broad categories such as susceptible or infected group, while 5 studies (23.8%) employed an agent-based modeling approach that simulate individual “agents” such as patients or staff. Additionally, 15 studies (71.4%) used stochastic models that include random variation in their simulation approach to reflect real-world transmission, 3 studies (14.3%) applied deterministic models which produces predictable outcomes during the simulation process, and 3 studies (14.3%) utilized hybrid models that blend predictable structure with random variation in their outcomes. The research articles identified originated from nine countries, with the majority conducted in the United States (40%) and Australia (28%).

For the second systematic review, a total of 2,826 studies were screened after searching four major databases: PubMed (722), CINAHL (963), Web of Science (759), and the Cochrane Library (382). A cross-reference search and additional reputable sources yielded 19 additional studies, which were also screened for inclusion eligibility. After removing 428 duplicates, followed by title and abstract screening and eligibility assessment, 7 studies met the inclusion criteria.

More information can be found in Figure 1a and 1b. Additional details on the search strategy for studies included in this review are available in the Supplementary Document attached.

### Basic reproduction number (R₀)

Five infection model studies provided estimates of the basic reproduction number (R₀) which is defined as the average number of secondary infections generated by one infectious case in a fully susceptible population. The basic reproduction number (R₀)—ranged from 0.28, suggesting limited hospital spread,^35^ to as high as 2.6, which implies sustained in-hospital transmission.^36^

It has been hypothesized that *C. difficile* transmission cannot be sustained in hospitals without continuous importation from the community. Other hypotheses emphasize the role of asymptomatic colonization in *C. difficile* transmission within hospital settings.^15,17^

A study estimated R₀ values to be 1.09 in the community, 1.11 in the general population, and 0.28 in hospital settings.^35^ Since R₀ was less than 1 in this hospital, this suggests that sustained hospital-based transmission alone may not account for ongoing CDI cases, supporting the hypothesis that infections may originate from community sources in this case and not solely hospital-acquired.^35^ In contrast, another study from our review reported the highest R₀ of 2.6 in a hospital setting, suggesting that *C. difficile* can continue to propagate within hospitals even in the absence of community importation.^36^ The other infection modeling studies found the R₀ to be 1.07,^37^ 0.57,^38^ 0.44, and 0.67.^39^

### Transmission coefficient

We identified 10 studies that reported transmission coefficients a measure of how efficiently a pathogen spreads; it reflects the likelihood that susceptible individuals become infected through contact with infectious individuals or contaminated environments. Most studies provided estimates for *C. difficile* transfer between patients, or from both patients and the healthcare environment. Reported transmission coefficient estimates ranged from 0.001 to 0.5, highlighting that the risk of *C. difficile* spread can vary substantially across hospital settings and infection control practices. Only one study quantified transmission specifically from contaminated fomites (environmental sources).^40^ See Table 2 for more details.

**Table 2.** Estimated transmission coefficients for *C. difficile* spread across different models and contexts

First author surname and year	Type and class of mathematical model	Parameter source(s)	Transmission coefficient (efficiency of pathogen spread)	Context/notes
Yakob 2014^41^	Deterministic and compartmental model	Published literature	0.5	General transmission coefficient
McLure 2019^35^	Stochastic and compartmental	Published literature	0.128 (0.071–0.164)	for colonized adults with disrupted gut flora (due to recent antibiotic exposure)
			0.019 (0.001–0.026)	for colonized adults with intact gut flora (no recent antibiotic exposure)
			0.064	for community from infants
Lanzas 2011^37^	Hybrid model and compartmental model	Observational data and published literature	0.007 (0.004–0.01)	General transmission context
McLure 2017^39^	Stochastic and compartmental	Published literature	0.09	General transmission context
Grigoras 2016^42^	Stochastic and compartmental	Published literature	0.1059	Susceptible with infected patients under contact precautions
			0.007	Susceptible with colonized on admission patients not under contact precautions
McLure 2018^43^	Stochastic and compartmental model	Published literature	0.09 (Range: 0.03–0.18)	General transmission context
Stephenson 2017^44^	Deterministic and compartmental	Observational data and published literature	0.007	Increased transmission coefficient
			0.0007	Average transmission coefficients
			0.00001	Transmission coefficients for asymptomatic carriers and diseased patients, respectively, per individual per day
McLure 2019^45^	Stochastic and compartmental	Published literature	0.128 (0.071–0.164)	for colonized adults with disrupted gut flora (due to recent antibiotic exposure)
			0.019 (0.001–0.026)	coefficient for colonized adults with intact gut flora (no recent antibiotic exposure)
Yakob 2015^46^	Stochastic and compartmental	Simulation and published literature	1–1.5	for hypervirulent; endemic strain
Van Kleef 2016^47^	Stochastic and agent-based	Observational data and published literature	0.0074	infected patients
			0.0037	Colonized patients

### Recovery rate and recurrence rate

Six studies^48–53^ provided information on recovery rates (rate at which infected individuals recover per unit time), which ranged from 0.099 to 0.21 per day, meaning on average 10%–21% of patients with CDI recover each day. The recurrence rate (rate at which a patient experiences another episode of CDI after initial clinical resolution) ranged from 0.13 to 0.3 per day, indicating that nearly one-third of patients who initially improve may relapse within days (Table 3).

**Table 3.** Estimates of recovery rate or/and the recurrence rate

First author surname and year	Type and class of mathematical model	Parameter source(s)	Recovery rates (per day)	Recurrence rates (per day)
Agnew 2023^48^	Stochastic and compartmental model	Published literature	0.21 (Range: 0.17–0.27)	–
Durham 2016^48^	Stochastic and compartmental	Observational data and published literature	0.099	0.13
McLure 2017^39^	Stochastic and compartmental	Published data	0.1	–
Maghdoori 2017^48^	Stochastic and compartmental	Observational data and published literature	0.2 (Range: 0.143–0.33)	–
Rhea 2020^48^	Stochastic and agent-based	Published literature	0.09426	0.1219
Toth 2020^48^	Stochastic and agent-based	Published literature	0.1	–
Lofgren 2014^48^	Stochastic and compartmental	Observational data and published literature	–	0.3

### Hospital discharge rate

We identified nine studies^38,41,44,54–60^ that estimated hospital discharge rates (Table 4). Reported values ranged from 0.04 to 0.2 per day. This broad range reflects variations in patient populations, healthcare settings, and modeling assumptions.

**Table 4.** Estimates of hospital discharge rates

First author surname and year	Type and class of mathematical model	Parameter source(s)	Hospital discharge rate (per day)
Agnew 2023^48^	Stochastic and compartmental model	Published literature	0.131
Chamchod 2019^38^	Hybrid (both deterministic and stochastic) and compartmental	Published literature	0.2
Lanzas 2011^37^	Hybrid (both deterministic and stochastic) and compartmental	Observational and published data	0.068 (for diseased patients) 0.15 (for susceptible and colonized patients)
Yakob 2013^57^	Deterministic and compartmental	Published literature	0.17
Sulyok 2021^40^	Hybrid (both deterministic and stochastic) and compartmental	Published literature	0.15 (Sensitivity range is 0.075, 0.225)
Yakob 2014^41^	Stochastic and agent-based	Published literature	0.17
Lofgren 2014^54^	Stochastic and compartmental	Observational data and published literature	0.04512
Stephenson 2017^44^	Deterministic and compartmental	Observational data and published literature	0.068
Maghdoori 2017^36^	Stochastic and compartmental	Published literature	0.17 (of hospital patients without symptomatic infection)

### Case fatality rates (CFR)

Nine studies reported case fatality rate (CFR) estimates (the proportion of infected individuals who die from the disease) used in their mathematical models. Reported CFR values ranged from 0.0000111 to 0.02 per day (Table 5). CFR values were reported per day to align with model time steps and are not directly comparable to clinical mortality metrics.

**Table 5.** Estimates of case fatality rates (CFR)

First author surname and year	Type and class of mathematical model	Parameter source(s)	Case fatality or death rate (per day)
Agnew 2023^48^	Stochastic and compartmental model	Published literature	0.007
McLure 2017^39^	Stochastic and compartmental	Published literature	0.0075
Yakob 2013^57^	Deterministic and compartmental	Published literature	0.02
Yakob 2014^41^	Deterministic and compartmental	Published literature	0.0012
Lofgren 2014^48^	Stochastic and compartmental	Observational data and Published literature	0.015
McLure 2019^35^	Stochastic and compartmental	Published literature	0.0000111
Maghdoori 2017^48^	Stochastic and compartmental	Published literature	0.0012 (0.001-0.01)
McLure 2019^45^	Stochastic and compartmental	Published literature	0.0000111 (general) 0.000184 (for elderly/immunosuppressed)
Yakob 2015^46^	Stochastic and compartmental	Simulation and published literature	0.0012

### Incubation period

We identified four studies that explicitly estimated the incubation period (time between infection and symptom onset) in their mathematical models.^47,61–63^ These studies reported incubation periods of 4, 5, 6, and 18 days, respectively.

### Ranking of high-touch surfaces

**Fig. 2.**
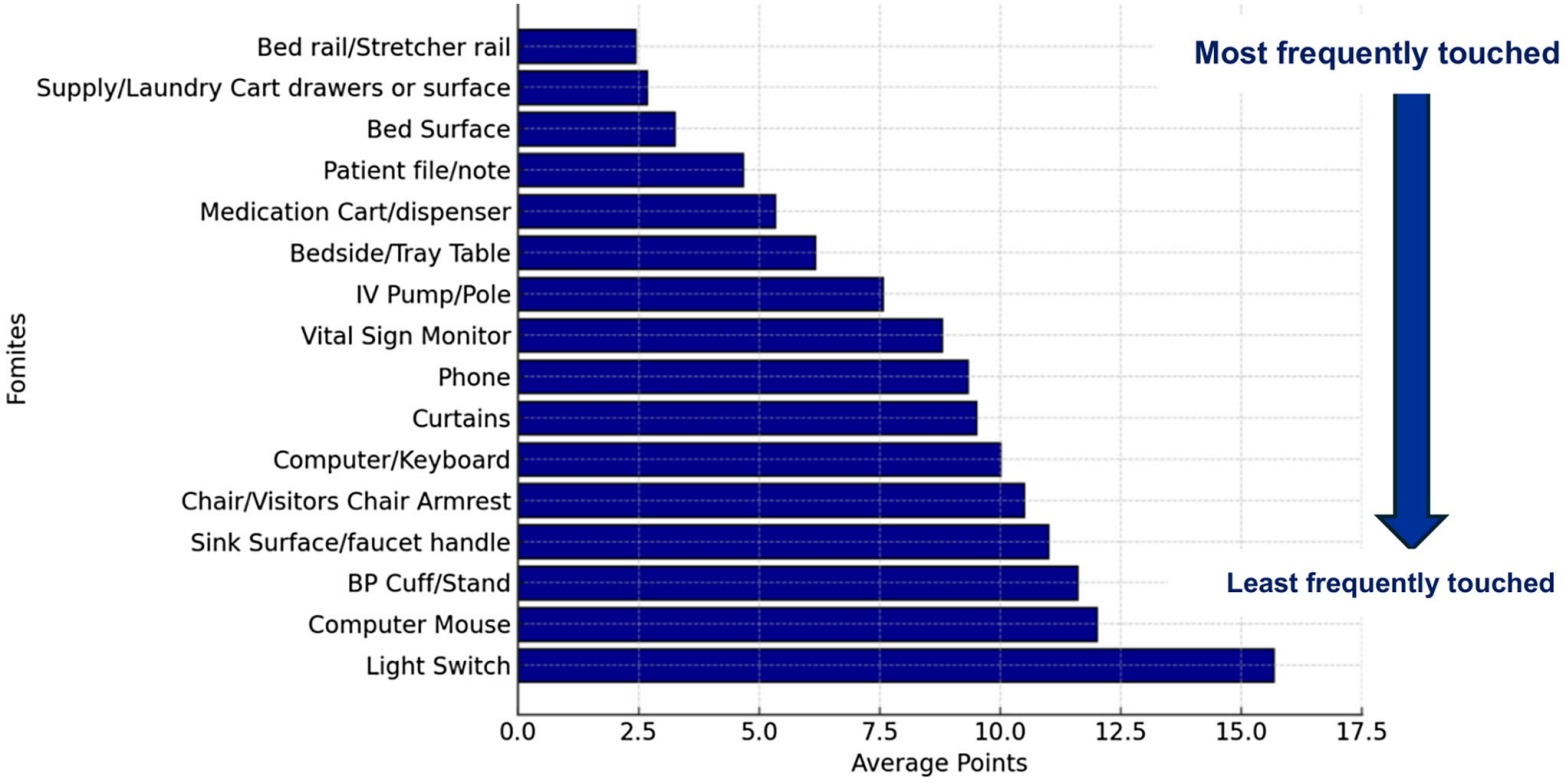
Ranking of high-touch surfaces across healthcare settings.

We ranked commonly mentioned HTS in the included studies based on their reported ranking in the primary studies across nine healthcare settings. Two studies out of the seven studies that reported on HTS, analyzed HTS in two different healthcare settings, contributing to the total of nine included settings.^64,65^ The most frequently touched fomites in each hospital setting received a score of 1, the second most frequently touched received a score of 2, and the least frequently touched received the highest numerical score. Overall, the most frequently touched fomite across all nine healthcare settings was the bed rail, with an average ranking score of 2.43. The other high-contact fomites in healthcare settings included bedside tables, supply carts, medication carts, patient notes, the patient’s body, computer keyboards, phones, computer mouses, bed surfaces, intravenous pumps, and vital signs monitors, among others (Fig. 2).

### Mutual-touch surfaces

We identified several mutually touched fomites based on evidence from the reviewed studies. Two studies provided quantitative data on mutual-touch surfaces.

Wang et al (2021) reported that the most commonly touched mutual surfaces were supply cart drawers, handwashing faucet handles, video translator machines, medication dispensers, supply cart surfaces, thermometers, scales, portable vital signs machines, and light switches, with contact frequencies of 17.1, 10.33, 3.0, 2.5, 2.0, 1.5, 1.0, and 0.4 contacts per hour, respectively.^64^

Cheng et al (2015) found that the most frequently touched mutual surfaces included the bedside rail, bedside table, patient body, patient file, linen, curtain, bed frame, and locker, with total recorded contact-episodes per hour of 13.6, 12.3, 9.4, 9.3, 6.1, 4.3, and 3.6, respectively.^66^

### Contact duration

None of the reviewed studies quantitatively measured contact duration, although they provided quantitative data on contact frequency for various fomites and surfaces.

### Assessment of heterogeneity and temporal variation in contact patterns

There was high heterogeneity among the included studies, as they used different measurement indices to quantify contact structures in healthcare settings. While three studies reported the total number or frequency of touches,^66–68^ two studies used “contacts per hour.”^64,66^ Huslage et al (2010) measured the “mean number of contacts per interaction,” Link et al (2016) reported the “mean frequency of touch,” and Suwantarat et al (2017) used “interactions per patient per hour” as a contact measurement.^65,69,70^ Additionally, two studies employed covert observational techniques, while the remaining five studies used direct observations.

Among the reviewed studies, only one study provided evidence of seasonal variations in contact frequency. The authors found that surface and patient contact episodes were more frequent during the summer compared to the winter. This finding was statistically significant (*P* = 0.002), with the odds of observing patient and/or surface contact activity in the summer being more than twice that of the winter period (CI: 0.269–0.738).^67^

## Discussion

Our study synthesizes quantitative evidence on essential parameters for CDI models which simulates how infection spreads in healthcare settings, including the basic reproduction number (R₀), incubation period, and recovery rate. Additionally, we provide a quantitative summary of HTSs in healthcare settings. As our systematic review provide evidence that a small number of fomites (surfaces) account for most contact episodes in healthcare settings, our findings suggest that targeted disinfection of frequently and mutually touched surfaces, such as bed rails and supply carts, may reduce environmental transmission and could be prioritized in resource-limited settings.

Our results also indicate a persistent paucity of studies that specifically estimate and/or explicitly report parameters for *C. difficile* infection models. Notably, different infection model types can produce different results because they make different assumptions. Compartmental models average outcomes across patients, while agent-based models capture variation and uncertainty, sometimes yielding wider ranges for intervention impact. Hence, model outputs should be interpreted cautiously and tailored to their specific setting.

Most of the modeling studies included in this review focused on hospital settings, particularly acute care facilities. Other settings, such as nursing homes and long-term care facilities, have been less frequently investigated. The lack of comprehensive surveillance data is a major limitation in expanding these models beyond acute care hospitals.^71^ Clinicians making decisions about infection prevention should note that infection models can help forecast the impact of enhanced cleaning strategies, patient isolation, or antibiotic stewardship interventions.^72^ Given the complexity of *C. difficile* transmission across different settings, key epidemiological parameters used in these models must be carefully verified and validated for each context.

Our study has several major strengths: the comprehensive synthesis of quantitative evidence on both *C. difficile* transmission parameters and the contribution of HTSs across healthcare settings, providing actionable insights for infection prevention planning. Also, we adhered to well-established systematic review methodologies, including guidelines from the Center for Reviews and Dissemination and PRISMA. Furthermore, we provide recommendations for improving mathematical models based on our findings.

One major limitation of current *C. difficile* modeling studies is the limited number of studies that provide R₀ estimates. When R₀ is estimated to be high (>1), suggesting sustained in-hospital transmission, hospital epidemiologists may prioritize stricter isolation protocols, enhanced cleaning, or environmental surveillance. In contrast, if R₀ is low (<1), indicating community importation, efforts may shift toward improving admission screening, diagnostic testing, or outpatient prevention strategies.

To translate these findings into practice, infection prevention teams should consider conducting environmental audits of high-touch and mutual-touch surfaces, especially in wards with high CDI incidence. Quantifying these contacts could help refine cleaning protocols and prioritize surfaces that pose the highest transmission risk. Our findings provide valuable insights to inform and improve future mathematical modeling studies on *C. difficile* transmission dynamics. By synthesizing key epidemiological parameters, this study facilitates easier identification and use of summarized quantitative estimates across different model, aiding researchers in developing more accurate models.

To conclude, accurate parameter estimates are critical for developing mathematical models that inform infectious disease control. Our study has summarized key parameters for modeling the acquisition and transmission of *C. difficile* across various settings and has provided quantitative evidence on human-environment contact patterns, particularly HTSs in healthcare settings. While gaps in evidence remain, modeling studies should expand beyond acute care hospitals. Our findings will strengthen infection control strategies by summarizing key infection modeling parameters to enable healthcare teams to simulate and prioritize the most effective interventions, optimize cleaning protocols, and refine *C. difficile* transmission models for more targeted CDI prevention.

## Supporting information

Olufadewa et al. supplementary material 1 Olufadewa et al. supplementary material

Olufadewa et al. supplementary material 2 Olufadewa et al. supplementary material

Olufadewa et al. supplementary material 3 Olufadewa et al. supplementary material

## Supplementary material

To view supplementary material for this article, please visit https://doi.org/10.1017/ice.2025.10302
