# Supplementary material for: Quantitative summarization of high-touch surfaces and epidemiological parameters of Clostridioides difficile acquisition and transmission for mathematical modeling: a systematic review
Source: Infect Control Hosp Epidemiol. 2025 Oct 15;46(12):1253–61. doi: 10.1017/ice.2025.10302 (PMC12779462; doi:10.1017/ice.2025.10302)
Supplement: Olufadewa et al. supplementary material 1 — Olufadewa et al. supplementary material [file S0899823X25103024sup001.docx]

**S1 Appendix. Search Strategy for Objective 1 on Epidemiology and Mathematical Modeling Parameters on *Clostridiodes difficile* modeling**

Date of Last Search: July 8^th^, 2023

Search Concepts

#1 - “Clostridiodes difficile” OR “Clostridium difficile” OR Clostridium OR Clostridiodes OR C. diff OR CDI

#2 - Mathematical OR Math* OR Model* OR “Mathematical Model” OR Modeling OR OR “Stochastic model” OR “Deterministic Model” OR “Agent-based Model” OR SEIR OR “SEIR Model” OR SEIRD OR “SEIR Model” OR “SIS Model” OR “SEIRD Model*” OR “SIR Model” OR “Individual-based Model” OR “Compartmental model”

#3 - “Transmission coefficient*” OR “Recovery rate*” OR “Recurrence rate*” OR “Incubation period*” OR “Basic reproduction number*” OR “Force of infection*” OR Saturation OR “Pathogen load*” OR “Hospital discharge rate*” OR “Case Fatality Rate*”

Combined Search Concept

(“Clostridiodes difficile” OR “Clostridium difficile” OR Clostridium OR Clostridiodes OR C. diff OR CDI) AND (Mathematical OR Math* OR Model* OR “Mathematical Model” OR Modeling OR OR “Stochastic model” OR “Deterministic Model” OR “Agent-based Model” OR SEIR OR “SEIR Model” OR SEIRD OR “SEIR Model” OR “SIS Model” OR “SEIRD Model*” OR “SIR Model” OR “Individual-based Model” OR “Compartmental model”) AND (“Transmission coefficient*” OR “Recovery rate*” OR “Recurrence rate*” OR “Incubation period*” OR “Basic reproduction number*” OR “Force of infection*” OR Saturation OR “Pathogen load*” OR “Hospital discharge rate*” OR “Case Fatality Rate*”)

Date Last Search Conducted: July 8^th^, 2023

PubMed

Number of papers found: 124

Search Term

(“Clostridiodes difficile” OR “Clostridium difficile” OR Clostridium OR Clostridiodes OR C. diff OR CDI) AND (Mathematical OR Math* OR Model* OR “Mathematical Model” OR Modeling OR “Stochastic model” OR “Deterministic Model” OR “Agent-based Model” OR SEIR OR “SEIR Model” OR SEIRD OR “SEIR Model” OR “SIS Model” OR “SEIRD Model*” OR “SIR Model” OR “Individual-based Model” OR “Compartmental model”) AND (“Transmission coefficient*” OR “Recovery rate*” OR “Recurrence rate*” OR “Incubation period*” OR “Basic reproduction number*” OR “Force of infection*” OR Saturation OR “Pathogen load*” OR “Hospital discharge rate*” OR “Case Fatality Rate*”)

Web of Science

Number of papers found: 163

#1-

“Clostridiodes difficile” OR “Clostridium difficile” OR Clostridium OR Clostridiodes OR C. diff OR CDI [all fields]

AND

#2-

Mathematical OR Math* OR Model* OR “Mathematical Model” OR Modeling OR “Stochastic model” OR “Deterministic Model” OR “Agent-based Model” OR SEIR OR “SEIR Model” OR SEIRD OR “SEIR Model” OR “SIS Model” OR “SEIRD Model*” OR “SIR Model” OR “Individual-based Model” OR “Compartmental model” [all fields]

AND

#3 –

“Transmission coefficient*” OR “Recovery rate*” OR “Recurrence rate*” OR “Incubation period*” OR “Basic reproduction number*” OR “Force of infection*” OR Saturation OR “Pathogen load*” OR “Hospital discharge rate*” OR “Case Fatality Rate*” [all fields]

CINAHL

Number of papers found: 18

Search Term

(“Clostridiodes difficile” OR “Clostridium difficile” OR Clostridium OR Clostridiodes OR C. diff OR CDI) AND (Mathematical OR Math* OR Model* OR “Mathematical Model” OR Modeling OR OR “Stochastic model” OR “Deterministic Model” OR “Agent-based Model” OR SEIR OR “SEIR Model” OR SEIRD OR “SEIR Model” OR “SIS Model” OR “SEIRD Model*” OR “SIR Model” OR “Individual-based Model” OR “Compartmental model”) AND (“Transmission coefficient*” OR “Recovery rate*” OR “Recurrence rate*” OR “Incubation period*” OR “Basic reproduction number*” OR “Force of infection*” OR Saturation OR “Pathogen load*” OR “Hospital discharge rate*” OR “Case Fatality Rate*”)

Cochrane Review

Number of papers found: 23

#1-

“Clostridiodes difficile” OR “Clostridium difficile” OR Clostridium OR Clostridiodes OR C. diff OR CDI [all fields]

AND

#2-

Mathematical OR Math* OR Model* OR “Mathematical Model” OR Modeling OR “Stochastic model” OR “Deterministic Model” OR “Agent-based Model” OR SEIR OR “SEIR Model” OR SEIRD OR “SEIR Model” OR “SIS Model” OR “SEIRD Model*” OR “SIR Model” OR “Individual-based Model” OR “Compartmental model” [all fields]

AND

#3 –

“Transmission coefficient*” OR “Recovery rate*” OR “Recurrence rate*” OR “Incubation period*” OR “Basic reproduction number*” OR “Force of infection*” OR Saturation OR “Pathogen load*” OR “Hospital discharge rate*” OR “Case Fatality Rate*” [all fields]

**S1 Appendix. Search Strategy for Objective 2 on identifying high touch fomites in HAIs**

Reporting of the search strategy

**Platform and databases searched:**

1. PubMed
2. Web of Science
3. CINAHL
4. Cochrane Database

**Date last search was conducted:** June 30, 2023

**Date Restriction:** we searched from database onset till the date of last search above.

**Language Restriction:** No language restriction

**Document Restriction:** No document restriction

**Summary of the search strategy:**

The search strategy was divided into four groups of keywords:

#1: Fomites

#2: Healthcare-associated infections

#3: Contact Pattern

#4: Healthcare Setting

The four groups were combined with the following logic: **#1 AND #2 AND #3 and #4**

**#1 (FOMITE)-** Fomite* OR table* OR Computer mouse OR Bed* OR Light Switch OR Door OR Door Handle OR Doorknobs OR Countertops OR Handrails OR Stretcher rails OR Fomites OR privacy curtain* OR Chair OR Armrest OR Supply cart OR Drawers OR Faucet OR Work table OR keyboard OR Bed rail* OR Computer OR Chair armrest OR Control panel* OR Note* OR Case note* OR IV drip OR Bed frame* OR Notes Trolley* OR Clothing* OR Mobile Phone* OR Gadgets OR Stethoscope* OR Endoscopes* OR Endocavity probe* OR vaginal probe* OR rectal probe OR Bronchoscope* OR Anesthesia equipment OR Respiratory therapy equipment OR Hand OR Patients Body OR Catheter OR Central Line* OR Peripheral Lines OR Surgical Instrument OR Urinary Catheter* OR Materials OR Biro OR video translator machine OR Wheelchairs OR Clamps OR Forceps OR Floor OR Ceiling OR Curtains OR Toilet OR Toilet Seats OR IV Pump Monitor* OR Keyboard* OR IV Tubings OR Intravenous Pump* OR Patients Body OR Patients Note OR Ventilator OR Sink)

**#2 (HEALTHCARE-ASSOCIATED INFECTIONS)-** "Clostridiodes difficile" OR "C. difficile" OR "MRSA" or "Methicillin Resistant Staphylococcus Aureus" OR VRE OR "Vancomycin Resistant Enterococcus" OR Norovirus* OR "Staphylococcus Aureus" OR "S. aureus" OR COVID-19 OR Sars-CoV-2 OR Coronavirus OR "Coronavirus Disease*" OR "Healthcare Associated Infection*" OR "Infectious Disease*" OR HAI OR HAIs OR HCAI OR HCAIs)

**#3 (CONTACT PATTERN)-** High-Touch OR Low-Touch OR "Frequently Touched" OR "High Touch" OR "High Touch surfaces" OR Critical OR Semi-Critical OR Non-Critical OR "Less Frequently Touched" OR Mutual-contact OR "Low Touch" OR "Low Touch Surfaces" OR Mutual-Touch OR "Mutual Contact" OR Contact OR "Contact Duration" OR "Duration of Contact" OR Duration OR "Contact Lasting" Or "Touch Lasting" OR "Touched For" OR "Contacted" OR Surface*

**#4 (HEALTHCARE SETTING)-** "Intensive Care Unit*" OR "Emergency Clinic*" OR "Patient Ward*" OR "Emergency Ward*" OR "Surgical Theatre*" OR "Medical Ward**" OR "Outpatient Clinic*" OR "Outpatient Ward*" OR "Accident and Emergency Unit*" OR "Geriatric Center*" OR "Geriatric Home*" OR "Elderly Person Home*" OR "Neonatal Intensive Care Unit" OR NICU OR "Hemodialysis Ward*" OR "Emergency Department*" OR "Operating Room*" OR Surgical-medical ward* OR "Acute Medical Ward*" OR "Acute Surgical Wards" OR "Acute Neurological Ward*" OR "Haemodialysis Facilit*" OR "Renal Unit*" OR "Admission Ward*" OR “In-patient Room*” OR "Hospital Room*"

**COMPLETE COMBINED SEARCH STRATEGY**

(Fomite* OR table* OR Computer mouse OR Bed* OR Light Switch OR Door OR Door Handle OR Doorknobs OR Countertops OR Handrails OR Stretcher rails OR Fomites OR privacy curtain* OR Chair OR Armrest OR Supply cart OR Drawers OR Faucet OR Work table OR keyboard OR Bed rail* OR Computer OR Chair armrest OR Control panel* OR Note* OR Case note* OR IV drip OR Bed frame* OR Notes Trolley* OR Clothing* OR Mobile Phone* OR Gadgets OR Stethoscope* OR Endoscopes* OR Endocavity probe* OR vaginal probe* OR rectal probe OR Bronchoscope* OR Anesthesia equipment OR Respiratory therapy equipment OR Hand OR Patients Body OR Catheter OR Central Line* OR Peripheral Lines OR Surgical Instrument OR Urinary Catheter* OR Materials OR Biro OR video translator machine OR Wheelchairs OR Clamps OR Forceps OR Floor OR Ceiling OR Curtains OR Toilet OR Toilet Seats OR IV Pump Monitor* OR Keyboard* OR IV Tubings OR Intravenous Pump* OR Patients Body OR Patients Note OR Ventilator OR Sink) AND ("Clostridiodes difficile" OR "C. difficile" OR "MRSA" or "Methicillin Resistant Staphylococcus Aureus" OR VRE OR "Vancomycin Resistant Enterococcus" OR Norovirus* OR "Staphylococcus Aureus" OR "S. aureus" OR COVID-19 OR Sars-CoV-2 OR Coronavirus OR "Coronavirus Disease*" OR "Healthcare Associated Infection*" OR "Infectious Disease*" OR HAI OR HAIs OR HCAI OR HCAIs) AND (High-Touch OR Low-Touch OR "Frequently Touched" OR "High Touch" OR "High Touch surfaces" OR Critical OR Semi-Critical OR Non-Critical OR "Less Frequently Touched" OR Mutual-contact OR "Low Touch" OR "Low Touch Surfaces" OR Mutual-Touch OR "Mutual Contact" OR Contact OR "Contact Duration" OR "Duration of Contact" OR Duration OR "Contact Lasting" Or "Touch Lasting" OR "Touched For" OR "Contacted" OR Surface*) AND ("Intensive Care Unit*" OR "Emergency Clinic*" OR "Patient Ward*" OR "Emergency Ward*" OR "Surgical Theatre*" OR "Medical Ward**" OR "Outpatient Clinic*" OR "Outpatient Ward*" OR "Accident and Emergency Unit*" OR "Geriatric Center*" OR "Geriatric Home*" OR "Elderly Person Home*" OR "Neonatal Intensive Care Unit" OR NICU OR "Hemodialysis Ward*" OR "Emergency Department*" OR "Operating Room*" OR Surgical-medical ward* OR "Acute Medical Ward*" OR "Acute Surgical Wards" OR "Acute Neurological Ward*" OR "Haemodialysis Facilit*" OR "Renal Unit*" OR "Admission Ward*" OR “In-patient Room*” OR "Hospital Room*")

**PUBMED**

Result gotten: **722**

**Complete Search Query:**

(Fomite* OR table* OR Computer mouse OR Bed* OR Light Switch OR Door OR Door Handle OR Doorknobs OR Countertops OR Handrails OR Stretcher rails OR Fomites OR privacy curtain* OR Chair OR Armrest OR Supply cart OR Drawers OR Faucet OR Work table OR keyboard OR Bed rail* OR Computer OR Chair armrest OR Control panel* OR Note* OR Case note* OR IV drip OR Bed frame* OR Notes Trolley* OR Clothing* OR Mobile Phone* OR Gadgets OR Stethoscope* OR Endoscopes* OR Endocavity probe* OR vaginal probe* OR rectal probe OR Bronchoscope* OR Anesthesia equipment OR Respiratory therapy equipment OR Hand OR Patients Body OR Catheter OR Central Line* OR Peripheral Lines OR Surgical Instrument OR Urinary Catheter* OR Materials OR Biro OR video translator machine OR Wheelchairs OR Clamps OR Forceps OR Floor OR Ceiling OR Curtains OR Toilet OR Toilet Seats OR IV Pump Monitor* OR Keyboard* OR IV Tubings OR Intravenous Pump* OR Patients Body OR Patients Note OR Ventilator OR Sink) AND ("Clostridiodes difficile" OR "C. difficile" OR "MRSA" or "Methicillin Resistant Staphylococcus Aureus" OR VRE OR "Vancomycin Resistant Enterococcus" OR Norovirus* OR "Staphylococcus Aureus" OR "S. aureus" OR COVID-19 OR Sars-CoV-2 OR Coronavirus OR "Coronavirus Disease*" OR "Healthcare Associated Infection*" OR "Infectious Disease*" OR HAI OR HAIs OR HCAI OR HCAIs) AND (High-Touch OR Low-Touch OR "Frequently Touched" OR "High Touch" OR "High Touch surfaces" OR Critical OR Semi-Critical OR Non-Critical OR "Less Frequently Touched" OR Mutual-contact OR "Low Touch" OR "Low Touch Surfaces" OR Mutual-Touch OR "Mutual Contact" OR Contact OR "Contact Duration" OR "Duration of Contact" OR Duration OR "Contact Lasting" Or "Touch Lasting" OR "Touched For" OR "Contacted" OR Surface*) AND ("Intensive Care Unit*" OR "Emergency Clinic*" OR "Patient Ward*" OR "Emergency Ward*" OR "Surgical Theatre*" OR "Medical Ward**" OR "Outpatient Clinic*" OR "Outpatient Ward*" OR "Accident and Emergency Unit*" OR "Geriatric Center*" OR "Geriatric Home*" OR "Elderly Person Home*" OR "Neonatal Intensive Care Unit" OR NICU OR "Hemodialysis Ward*" OR "Emergency Department*" OR "Operating Room*" OR Surgical-medical ward* OR "Acute Medical Ward*" OR "Acute Surgical Wards" OR "Acute Neurological Ward*" OR "Haemodialysis Facilit*" OR "Renal Unit*" OR "Admission Ward*" OR “In-patient Room*” OR "Hospital Room*")

**WEB OF SCIENCE**

Result gotten: **759**

Complete Search Query:

(Fomite* OR table* OR Computer mouse OR Bed* OR Light Switch OR Door OR Door Handle OR Doorknobs OR Countertops OR Handrails OR Stretcher rails OR Fomites OR privacy curtain* OR Chair OR Armrest OR Supply cart OR Drawers OR Faucet OR Work table OR keyboard OR Bed rail* OR Computer OR Chair armrest OR Control panel* OR Note* OR Case note* OR IV drip OR Bed frame* OR Notes Trolley* OR Clothing* OR Mobile Phone* OR Gadgets OR Stethoscope* OR Endoscopes* OR Endocavity probe* OR vaginal probe* OR rectal probe OR Bronchoscope* OR Anesthesia equipment OR Respiratory therapy equipment OR Hand OR Patients Body OR Catheter OR Central Line* OR Peripheral Lines OR Surgical Instrument OR Urinary Catheter* OR Materials OR Biro OR video translator machine OR Wheelchairs OR Clamps OR Forceps OR Floor OR Ceiling OR Curtains OR Toilet OR Toilet Seats OR IV Pump Monitor* OR Keyboard* OR IV Tubings OR Intravenous Pump* OR Patients Body OR Patients Note OR Ventilator OR Sink) AND ("Clostridiodes difficile" OR "C. difficile" OR "MRSA" or "Methicillin Resistant Staphylococcus Aureus" OR VRE OR "Vancomycin Resistant Enterococcus" OR Norovirus* OR "Staphylococcus Aureus" OR "S. aureus" OR "Healthcare Associated Infection*" OR "Infectious Disease*" OR HAI OR HAIs OR HCAI OR HCAIs) AND (High-Touch OR Low-Touch OR "Frequently Touched" OR "High Touch" OR "High Touch surfaces" OR Critical OR Semi-Critical OR Non-Critical OR "Less Frequently Touched" OR Mutual-contact OR "Low Touch" OR "Low Touch Surfaces" OR Mutual-Touch OR "Mutual Contact" OR Contact OR "Contact Duration" OR "Duration of Contact" OR Duration OR "Contact Lasting" Or "Touch Lasting" OR "Touched For" OR "Contacted" OR Surface*) AND ("Intensive Care Unit*" OR "Emergency Clinic*" OR "Patient Ward*" OR "Emergency Ward*" OR "Surgical Theatre*" OR "Medical Ward**" OR "Outpatient Clinic*" OR "Outpatient Ward*" OR "Accident and Emergency Unit*" OR "Geriatric Center*" OR "Geriatric Home*" OR "Elderly Person Home*" OR "Neonatal Intensive Care Unit" OR NICU OR "Hemodialysis Ward*" OR "Emergency Department*" OR "Operating Room*" OR Surgical-medical ward* OR "Acute Medical Ward*" OR "Acute Surgical Wards" OR "Acute Neurological Ward*" OR "Haemodialysis Facilit*" OR "Renal Unit*" OR "Admission Ward*" OR “In-patient Room*” OR "Hospital Room*")

**COCHCHRANE LIBRARY (COCHRANE REVIEW)**

Result gotten: **382**

Complete Search Query

#1: Fomite* OR table* OR Computer mouse OR Bed* OR Light Switch OR Door OR Door Handle OR Doorknobs OR Countertops OR Handrails OR Stretcher rails OR Fomites OR privacy curtain* OR Chair OR Armrest OR Supply cart OR Drawers OR Faucet OR Work table OR keyboard OR Bed rail* OR Computer OR Chair armrest OR Control panel* OR Note* OR Case note* OR IV drip OR Bed frame* OR Notes Trolley* OR Clothing* OR Mobile Phone* OR Gadgets OR Stethoscope* OR Endoscopes* OR Endocavity probe* OR vaginal probe* OR rectal probe OR Bronchoscope* OR Anesthesia equipment OR Respiratory therapy equipment OR Hand OR Patients Body OR Catheter OR Central Line* OR Peripheral Lines OR Surgical Instrument OR Urinary Catheter* OR Materials OR Biro OR video translator machine OR Wheelchairs OR Clamps OR Forceps OR Floor OR Ceiling OR Curtains OR Toilet OR Toilet Seats OR IV Pump Monitor* OR Keyboard* OR IV Tubings OR Intravenous Pump* OR Patients Body OR Patients Note OR Ventilator OR Sink

#2: Clostridiodes difficile OR C. difficile OR MRSA OR Methicillin Resistant Staphylococcus Aureus OR VRE OR Vancomycin Resistant Enterococcus OR Norovirus* OR Staphylococcus Aureus OR S. aureus OR COVID-19 OR Sars-CoV-2 OR Coronavirus OR Coronavirus Disease* OR Healthcare Associated Infection* OR Infectious Disease* OR HAI OR HAIs OR HCAI OR HCAIs

#3: High-Touch OR Low-Touch OR "Frequently Touched" OR "High Touch" OR "High Touch surfaces" OR Critical OR Semi-Critical OR Non-Critical OR "Less Frequently Touched" OR Mutual-contact OR "Low Touch" OR "Low Touch Surfaces" OR Mutual-Touch OR "Mutual Contact" OR Contact OR "Contact Duration" OR "Duration of Contact" OR Duration OR "Contact Lasting" Or "Touch Lasting" OR "Touched For" OR "Contacted" OR Surface*

#4: Intensive Care Unit* OR Emergency Clinic* OR "Patient Ward* OR Emergency Ward* OR Surgical Theatre* OR Medical Ward* OR "Outpatient Clinic* OR Outpatient Ward* OR Accident and Emergency Unit* OR Geriatric Center* OR Geriatric Home* OR Elderly Person Home* OR Neonatal Intensive Care Unit OR NICU OR Hemodialysis Ward* OR Emergency Department* OR Operating Room* OR Surgical-medical ward* OR Acute Medical Ward* OR Acute Surgical Wards OR Acute Neurological Ward* OR Haemodialysis Facilit* OR Renal Unit* OR Admission Ward* OR In-patient Room* OR Hospital Room*

**CINAHL**

Results gotten: **963**

Complete Search Query

(Fomite* OR table* OR Computer mouse OR Bed* OR Light Switch OR Door OR Door Handle OR Doorknobs OR Countertops OR Handrails OR Stretcher rails OR Fomites OR privacy curtain* OR Chair OR Armrest OR Supply cart OR Drawers OR Faucet OR Work table OR keyboard OR Bed rail* OR Computer OR Chair armrest OR Control panel* OR Note* OR Case note* OR IV drip OR Bed frame* OR Notes Trolley* OR Clothing* OR Mobile Phone* OR Gadgets OR Stethoscope* OR Endoscopes* OR Endocavity probe* OR vaginal probe* OR rectal probe OR Bronchoscope* OR Anesthesia equipment OR Respiratory therapy equipment OR Hand OR Patients Body OR Catheter OR Central Line* OR Peripheral Lines OR Surgical Instrument OR Urinary Catheter* OR Materials OR Biro OR video translator machine OR Wheelchairs OR Clamps OR Forceps OR Floor OR Ceiling OR Curtains OR Toilet OR Toilet Seats OR IV Pump Monitor* OR Keyboard* OR IV Tubings OR Intravenous Pump* OR Patients Body OR Patients Note OR Ventilator OR Sink) AND ("Clostridiodes difficile" OR "C. difficile" OR "MRSA" or "Methicillin Resistant Staphylococcus Aureus" OR VRE OR "Vancomycin Resistant Enterococcus" OR Norovirus* OR "Staphylococcus Aureus" OR "S. aureus" OR "Healthcare Associated Infection*" OR "Infectious Disease*" OR HAI OR HAIs OR HCAI OR HCAIs) AND (High-Touch OR Low-Touch OR "Frequently Touched" OR "High Touch" OR "High Touch surfaces" OR Critical OR Semi-Critical OR Non-Critical OR "Less Frequently Touched" OR Mutual-contact OR "Low Touch" OR "Low Touch Surfaces" OR Mutual-Touch OR "Mutual Contact" OR Contact OR "Contact Duration" OR "Duration of Contact" OR Duration OR "Contact Lasting" Or "Touch Lasting" OR "Touched For" OR "Contacted" OR Surface*) AND ("Intensive Care Unit*" OR "Emergency Clinic*" OR "Patient Ward*" OR "Emergency Ward*" OR "Surgical Theatre*" OR "Medical Ward**" OR "Outpatient Clinic*" OR "Outpatient Ward*" OR "Accident and Emergency Unit*" OR "Geriatric Center*" OR "Geriatric Home*" OR "Elderly Person Home*" OR "Neonatal Intensive Care Unit" OR NICU OR "Hemodialysis Ward*" OR "Emergency Department*" OR "Operating Room*" OR Surgical-medical ward* OR "Acute Medical Ward*" OR "Acute Surgical Wards" OR "Acute Neurological Ward*" OR "Haemodialysis Facilit*" OR "Renal Unit*" OR "Admission Ward*" OR “In-patient Room*” OR "Hospital Room*")
